# ^13^C-Metabolic Flux Analysis: An Accurate Approach to Demystify Microbial Metabolism for Biochemical Production

**DOI:** 10.3390/bioengineering3010003

**Published:** 2015-12-25

**Authors:** Weihua Guo, Jiayuan Sheng, Xueyang Feng

**Affiliations:** Department of Biological Systems Engineering, Virginia Polytechnic Institute and State University, Blacksburg, VA 24061, USA; gweihua2@vt.edu (W.G.); jysheng@vt.edu (J.S.)

**Keywords:** Bottleneck, isotope, cofactor imbalance, cell metabolism, synthetic biology, biofuels

## Abstract

Metabolic engineering of various industrial microorganisms to produce chemicals, fuels, and drugs has raised interest since it is environmentally friendly, sustainable, and independent of nonrenewable resources. However, microbial metabolism is so complex that only a few metabolic engineering efforts have been able to achieve a satisfactory yield, titer or productivity of the target chemicals for industrial commercialization. In order to overcome this challenge, ^13^C Metabolic Flux Analysis (^13^C-MFA) has been continuously developed and widely applied to rigorously investigate cell metabolism and quantify the carbon flux distribution in central metabolic pathways. In the past decade, many ^13^C-MFA studies have been performed in academic labs and biotechnology industries to pinpoint key issues related to microbe-based chemical production. Insightful information about the metabolic rewiring has been provided to guide the development of the appropriate metabolic engineering strategies for improving the biochemical production. In this review, we will introduce the basics of ^13^C-MFA and illustrate how ^13^C-MFA has been applied via integration with metabolic engineering to identify and tackle the rate-limiting steps in biochemical production for various host microorganisms

## 1. Introduction

Using microorganisms to produce various chemicals from renewable resources could be environmentally friendly and reduce strong dependence on petroleum. Recently, with the rapid development of metabolic engineering and synthetic biology, a wide range of bulk chemicals [1,2,3,4], biofuels [5,6,7,8,9], and drugs [10,11,12,13,14,15,16] from renewable feedstock have been produced by many industrial microorganisms such as *Escherichia coli* [17,18,19,20,21,22] and *Saccharomyces cerevisiae* [23,24,25,26]*.* However, only a few of these biosynthesized chemicals are able to be industrially commercialized due to low production levels with unsatisfactory titers, yields, and productivities [27,28]. Therefore, it is pivotal to develop novel strategies in metabolic engineering to improve microbe-based chemical production.

One of the main reasons for the low production level of engineered microorganisms is the high complexity of cell metabolism[28]. Microbial production of chemicals is more than the enzymatic conversion of the precursors to the products. Instead, to achieve the production of target chemicals at a high level, controls over microbial metabolism must coordinate the carbon flux [29,30], cofactor supply [31,32,33], cell maintenance [10,34,35], as well as other factors [36,37,38,39]. In general, many of the metabolic engineering strategies adopted to manipulate microbial metabolism for biochemical production only focus on a few known challenges (e.g., poor gene expression). However, attempting to overcome these challenges could result in new problems in host cells (e.g., metabolic burden) and hence prevent the microorganisms from achieving high-level chemical production. The lack of knowledge on such complex behavior of microbial physiology presents one of the most significant issues in improving the microbe-based chemical production.

To demystify the complex metabolic rewiring of engineered microorganisms and more importantly, derive the appropriate strategies to engineer microorganisms for better biochemical production, a technology named ^13^C-Metabolic Flux Analysis (^13^C-MFA) has been in development since the 1990s [40,41,42,43,44,45]. Basically, in ^13^C-MFA, carbon isotopes have been used to trace the cell metabolism. The carbon flux distributions in metabolic network of microorganisms can be determined using computational algorithms with the development of a metabolic model and the measurement of ^13^C-labeling patterns of the key metabolites [40,41,46,47,48,49]. By comparing variations of metabolic fluxes among different engineered microorganisms, the key issues, such as the bottleneck pathway, could often be revealed and hence guide the metabolic engineers to develop more appropriate strategies [35,50,51,52,53,54,55] for further improvement of chemical production. In the past decade, we have witnessed that numerous valuable biological insights pinpointed by ^13^C-MFA successfully helped to enhance the microbial production of chemicals [30,34,51,53,56,57]. Therefore, ^13^C-MFA has been widely considered as one of the most important tools to diagnose complex microbial metabolism and develop novel metabolic engineering strategies [29,30,32,34,35,53].

In this review, we aim to summarize the integrated tactics of ^13^C-MFA and metabolic engineering from cases that attempted to improve microbe-based chemical production in the past decade. We will first briefly introduce the techniques of ^13^C-MFA, and then categorize studies that integrated ^13^C-MFA with metabolic engineering in different industrial microorganisms, including (1) *Saccharomyces cerevisiae*; (2) *Escherichia coli*; (3) *Bacillus subtilis*; (4) *Corynebacterium glutamicum*; and (5) other microorganisms. Finally, we envision the emerging areas where breakthroughs of ^13^C-MFA could potentially promote rational metabolic engineering for improved microbe-based chemical production in future.

## 2. Techniques of ^13^C Metabolic Flux Analysis

**Figure 1:**
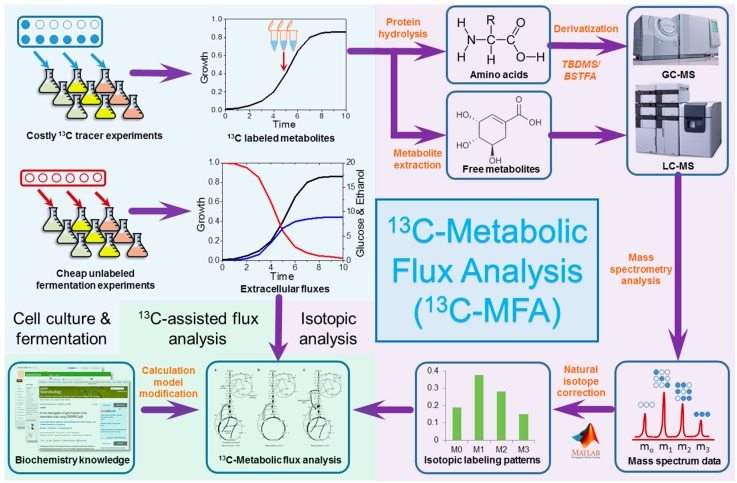
The scheme of ^13^C-Metabolic Flux Analysis (^13^C-MFA).

The techniques of ^13^C-MFA have been developed for more than two decades [40,41,42,43,44,45]. The development of advanced mathematical algorithms and high-throughput mass spectrometry technology enables accurate and quantitative analysis of metabolic fluxes for various microorganisms. Since several protocols have been published to thoroughly describe the procedures for both model and non-model organisms [46,58], in this review we will only provide a concise introduction on ^13^C-MFA, which includes three steps in general: cell cultivation, isotopic analysis of metabolites, and ^13^C-assisted pathway and flux analysis (Figure 1).

Cell culture on ^13^C-labeled carbon substrate is the first step of ^13^C-MFA and plays a vital role for the entire flux analysis. The choice of ^13^C-labeled substrate is a key factor for ^13^C-MFA, which depends on the choice of the target microorganism and the objectives of the experiment. In general, to elucidate the flux distribution accurately, a well-studied glucose mixture, *i.e.*, 80% [1-^13^C] and 20% [U-^13^C] glucose (*w*/*w*), has often been used to guarantee that high ^13^C abundance will be observed in various metabolites [46,59]. On the other hand, pure and singly labeled carbon substrate is more suitable to discover novel pathways because it is easier to trace labeled carbons in intermediates [60]. A strictly minimal medium with only the selected ^13^C-labeled substrate as the sole carbon source is often required for the ^13^C-labeling experiments. Two culture modes, *i.e.*, batch mode and chemostat mode, are most often used for ^13^C-MFA to reach the required steady states for sampling, namely, metabolic and isotopic steady states, in which the concentration and isotopic labeling of intracellular metabolites are both constant. Next, cell biomass and culture medium will be used for further isotopic analysis.

The measurement of ^13^C-labeling in metabolites is often achieved by using mass spectrometry, such as gas chromatography–mass spectrometry (GC-MS) and liquid chromatography-mass spectrometry (LC-MS). When using GC-MS for isotopic analysis, a derivatization process using TBDMS or BSTFA as the derivatization agents is always required to render molecules (e.g., over-produced chemicals [61] and proteinogenic amino acids) volatile enough to be analyzed by GC-MS. When using LC-MS for isotopic analysis, metabolites with trace amounts or high instabilities can be directly analyzed because of the high sensitivity of LC-MS. Since the effects of naturally labeled isotopes cannot be ignored when analyzing the ^13^C-labeling of metabolites, systematic correction of such natural isotopic effects is taken to generate the isotopic distributions e.g., mass distribution vector (MDV), for the metabolites of interest by using several well-established algorithms [62,63,64]. Such isotopic distributions, *i.e.*, MDV, will then be used for the pathway and flux analysis [46].

Based on the MDV of various metabolites, the metabolic behaviors of microorganisms can be elucidated both qualitatively (*i.e.*, pathway analysis) and quantitatively (*i.e.*, flux analysis). On one hand, the ^13^C-assisted pathway analysis often aims to answer whether or not a metabolic pathway is active in non-model microorganisms by tracking the ^13^C-labeling patterns (*i.e.*, MDVs) in key metabolites and the fate of biomolecule synthesis in these denoted biochemical pathways. On the other hand, the ^13^C-assisted flux analysis aims to quantify the carbon fluxes in multiple metabolic pathways by simulating the ^13^C-labeling patterns (*i.e.*, MDVs) in key metabolites and searching for the “real” metabolic fluxes that could lead to the best fit of the measured MDV. In short, while ^13^C-assisted pathway analysis is suitable for pathway discovery in non-model microorganisms, ^13^C-assisted flux analysis is more useful in identifying the metabolic rewiring in industrial microorganisms.

**Figure 2:**
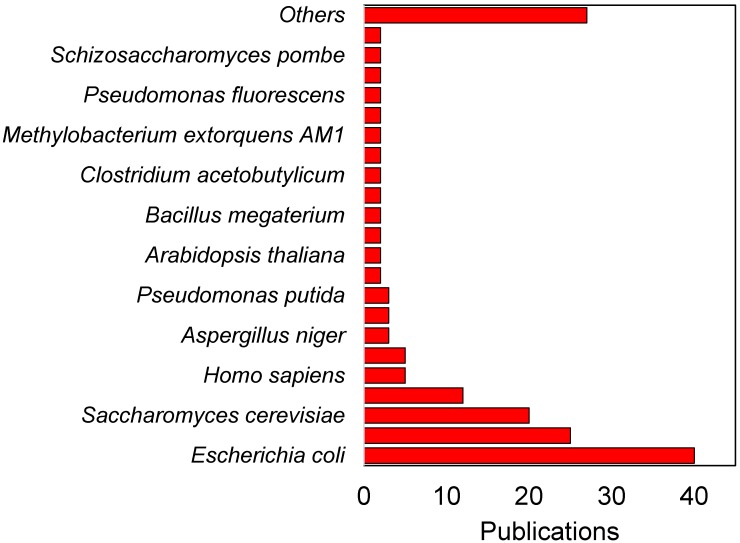
Summary of current ^13^C-MFA studies on different organisms.

In the past decade, ^13^C-assisted flux analysis has been widely applied to uncover the central metabolisms of various microorganisms. According to a curated database [64] that was developed to collect the metabolic fluxes investigated by ^13^C-MFA, over 500 cases of metabolic flux analysis have been accomplished so far for 36 organisms (Figure 2). Most of the ^13^C-MFA studies focus on investigating metabolism of *E.coli* and *S. cerevisiae*. However, there is a trend that other industrial microorganisms, such as *Clostridium* and *Cyanobacteria*, will initiate more ^13^C-MFA studies due to their importance in biochemical production and relatively less well-known cell metabolism. Also, with the wide application of ^13^C-MFA, many ^13^C-MFA software packages, such as OpenFLUX2 [65], 13CFLUX2 [66], Metran [67], INCA [68], FiatFLUX [69], and Biomet Toolbox 2.0 [70], have been developed with highly efficient mathematical algorithms (e.g., elementary metabolite unit, EMU [67,71]) to calculate carbon fluxes in various metabolic networks (Table 1). As a result, some of the difficulties, especially the computational load, of ^13^C-MFA have been dramatically decreased [71]. It is reasonable to believe that the number of ^13^C-MFA studies could increase by orders of magnitudes in the next decade or two.

## 3. Integrating ^13^C Metabolic Flux Analysis and Metabolic Engineering for Different Industrial Microorganisms

The ultimate goal of metabolic engineering is to design and build engineered biological systems that can produce chemicals, materials, food, and drugs at high yield using the appropriate microorganisms [72]. However, the lack of fundamental understanding of cellular responses during industrial bioprocesses often prevents metabolic engineers from achieving satisfactory goals in biochemical production. In the past decade, ^13^C-MFA has been widely used to provide insightful information on metabolism of various microorganisms, thus helping metabolic engineers to successfully improve biochemical production. In the following sections, we have summarized recent successes on integrating ^13^C-MFA and metabolic engineering (Table 2) based on different host organisms: (1) *Saccharomyces cerevisiae*; (2) *Escherichia coli*; (3) *Bacillus subtilis*; (4) *Corynebacterium glutamicum*; and (5) other host microorganisms.

**Table 1:** Summary of ^13^C-MFA software.

Name	Capabilities	Labeled Pattern	Key Solver (Algorithm)	Platform	Developer
13CFLUX2 [66]	Steady-state ^13^C-MFA	EMU [67] ^d^	IPOPT	UNIX/Linux	Wiechert’s group
Metran [67]	Steady-state ^13^C-MFA	EMU	*fmincon*	MATLAB	Antoniewicz’s group
FIA [177]	Steady-state ^13^C-MFA	Fluxomer	SNOPT [178]	UNIX/Linux	Young’s group
influx_s [179]	Steady-state ^13^C-MFA	Cumomer	NLSIC [179]	UNIX/Linux	Portais’s group
C13 [180]	Steady-state ^13^C-MFA	SFL [181] ^e^	*fmincon*	MATLAB	Nielsen’s group
OpenFLUX2 [65]	Steady-state ^13^C-MFA with PLEs ^a^	EMU	*fmincon*	MATLAB	Mashko’s group
FiatFLUX [69]	METAFoRA ^b^, steady-state ^13^C-MFA	MDV ^f^	*fmincon*	MATLAB	Sauer’s group
INCA [68]	Steady-state ^13^C-MFA and INST-^13^C-MFA ^c^	EMU	Customized Differential Equation Solver [71]	MATLAB	Young’s group
OpenMebius [182]	Steady-state ^13^C-MFA and INST-^13^C-MFA ^c^	EMU	*Levenberg-Marquardt* method [183]	MATLAB	Shimizu’s group

^a^ PLEs: Parallel isotopic experiments; ^b^ METAFoRA: Metabolic flux ratio analysis; ^c^ INST-^13^C-MFA: isotopic nonstationary ^13^C-metabolic flux analysis; ^d^ EMU: elementary metabolite unit; ^e^ SFL: summed fractional labeling; ^f^ MDV: mass distribution vectors.

**Table 2:** Summary of synergistic tactics of ^13^C-MFA and metabolic engineering ^a^.

Organism	Key Issues in Metabolic Engineering	Final Product (Objective)	Major Results of ^13^C-MFA	Strategies of Metabolic Engineering	Results of Metabolic Engineering
*S. cerevisiae*	Bottleneck step: Cytosolic acetyl-CoA supply	n-Butanol	Activated pyruvate bypass pathway [86]	Overexpress heterologous cyto-PDH [50], ACS, and pyruvate bypass pathway. Inactivate ADH and GPD [50].	300% increase of n-butanol production [50]
	Bottleneck step: Cytosolic acetyl-CoA supply	Isoprenoid-derived drugs [184]	Activated pyruvate bypass pathway [86]	Overexpress ALD [184], ACS	70% increase of amorphadiene production [184].
	Bottleneck step: Cytosolic acetyl-CoA supply	Various industrially relevant products	Activated parallel PHK pathway [51]	Introduce heterologous phosphoketolase pathway [51]	~10% increase of the acetyl-CoA supply independent with EMP pathwayb
	Bottleneck step: Pentose phosphate pathway	Shikimic acid Muconic acid	Activated shikimic acid synthesis pathway. Activated glycolysis replenishment from PP pathway.	Overexpress aro1, aro4, and tkl. Integrate with different host strains.	600% increase of shikimic acid production 2400% increase of munconic acid production [82]
*S. cerevisiae*	Cofactor imbalance issue	Ethanol (Xylose utilization) [35]	Activated oxidative PP pathway and TCA cycle [35]	Alternate the cofactor specificity of XR to NADH [90,91,92,94]. Alternate the cofactor specificity of XDH to NADP^+^ [99,100]	~56% increase the xylose consumption [91] ~40% increase of the ethanol production [94] ~50% decrease of the xylitol production [94]
*S. cerevisiae*	High maintenance energy	S-Adenosyl-L-methionine [34]	Active TCA cycle	Use low copy plasmids Proper promoter selection Nutrient medium optimization Co-substrate culture [185]	To be validated
	High maintenance energy	Xylose utilization [35]	Active TCA cycle		
*S. cerevisiae*	Stress response: Furfural [38]	Growth (Survival)	Activated PP pathway (NADPH supply)	Overexpress NADPH-dependent oxireductase in parent strain.	More than 20% decrease of biomass production [38]
*E. coli*	Bottleneck step: Cytosolic acetyl-CoA supply, reduction power supply.	Fatty acid and fatty acid derived chemicals [56,57]	600% increase of fatty acid synthesis pathway, increased PP, ED, and anaplerotic pathways. Decrease of PDC pathway.	Overexpress ACC, ACL, and relevant FASs. Knock out downstream FADs.	200% increase of fatty acid production
*E. coli* [186]	Cofactor imbalance issue	NADPH-dependent compounds [52,93,117] (Lycopene, fatty acid, *etc.*)	Insufficient NADPH supply from PP pathways Active transdehydrogenase pathway	Overexpress G6PDH [114,115,116] or transhydrogenase [120,121]. Knock out pgi [119].	~100% increase of lycopene production [52]
*E. coli*	Stress response: Octanoic acid [37]	Growth (Survival)	Repressed TCA cycle and PDH pathway. Deficient NAD+ supply. Activated PDC pathway	Decrease the cofactor (NADH/NAD+) sensitivity of particular enzymes. Overexpress the proteins of electron transport chain. Add other electron acceptor.	~50% increase of growth rate [37]
	Stress response: Super-oxidative (paraquat induced)	Growth (Survival)	Increased NADPH supply from oxidative PP pathway Repressed NADH production by diverting PDH pathway and TCA cycle to pyruvate bypass pathway and glyoxylate cycle, respectively	*Suggested strategies:*	To be validated
				Overexpress zwf gene to increase the supply of NADPH Overexpress the gene encoding transhydrogenase to increase the supply of NADPH	
*B. subtilis*	Bottleneck step: biosynthesis pathways [126]	Riboflavin	Highly activated PP pathway (precursors and NADPH supply) [126,130] Highly activated anaplerotic pathways (NADH supply) [126,127]	*Suggested strategies:*	To be validated
				Modification in biosynthesis pathways (e.g., overexpress the gene encoding the key enzymes for synthesis)	
*B. subtilis*	High maintenance energy [128,129]	Riboflavin	Increased TCA cycle and ATP requirements	Similar as that used for tackling high maintenance energy of S. cerevisiae	To be validated
*C. glutamicum*	Cofactor imbalance issue	L-lysine	Activated PP pathway (NADPH supply)	Overexpress the enzymes in PP pathway [136,137] Overexpress 1, 6-bisphosphatase [138] Alternate cofactor specificity of GAPDH [139]	~50% increase of the L-lysine production without the deficient growth [139]
	Cofactor imbalance issue	L-valine [53]	Activated PP pathway (NADPH supply)	Overexpress *E. coli* transhydrogenase	More than 200% increase of L-valine titer [53]
*P. pastoris*	High maintenance energy	*R. oryzae* lipase [185]	Active TCA cycle	Nutrient medium optimization [35] Co-substrate culture [185]	~35% increase of SAM production
*A. niger*	Cofactor imbalance issues	Fructofuranosidase [151]	Increased oxidative PP pathway (NADPH supply) [152] Increased malic enzyme pathway (NADPH supply) Decreased TCA cycle	*Suggested strategies:*	To be validated
				Increase the NADPH supply by heterologous pathways (transhydrogenase)	
*P. chrysogenum*	Cofactor imbalance issues[153]	Penicillin-G	Insufficient NADPH supply for penicillin production when additional NADPH is required	*Suggested strategies:*	To be validated
				Increase the NADPH supply (e.g., upregulate the G6P dehydrogenase or introduce transhydrogenase pathway)	
*R. palustris*	Cofactor imbalance issues [154]	Hydrogen	Shift of glyoxylate cycle to TCA cycle (NADH supply)	*Suggested strategies: *	To be validated
				Increase the NADH supply (e.g., overexpressing the NADH producing enzymes, or repress G6P dehydrogenase or isocitrate lyase)	
*B. succiniciproducens*	Bottleneck steps in precursor supply [155]	Succinate	Identify competitive pathways of precursor supply	Knock out the competitive genes for the identified pathways	~45% increase of succinate yield [155]

^a^ Abbreviations: ACC: Acetyl-CoA carboxylase, ACL: ATP citrate lyase, ACS: Acetyl-CoA synthetase, ADH: Alcohol dehydrogenase, ALD: Acetaldehyde dehydrogenase, ED: Entner-Doudoroff pathway, FAS: Fatty acid synthesis enzymes, FAD: Fatty acids degradation enzymes, G6PDH: G6P dehydrogenase, GAPDH: Glyceraldehyde 3-phosphate dehydrogenase, PDC: Pyruvate Decarboxylase, PDH: Pyruvate dehydrogenase, PP: Pentose phosphate pathway, XR: Xylose reductase, XDH: Xylitol dehydrogenase; ^b^ This value is estimated by flux values.

### 3.1. Saccharomyces Cerevisiae

**Figure 3:**
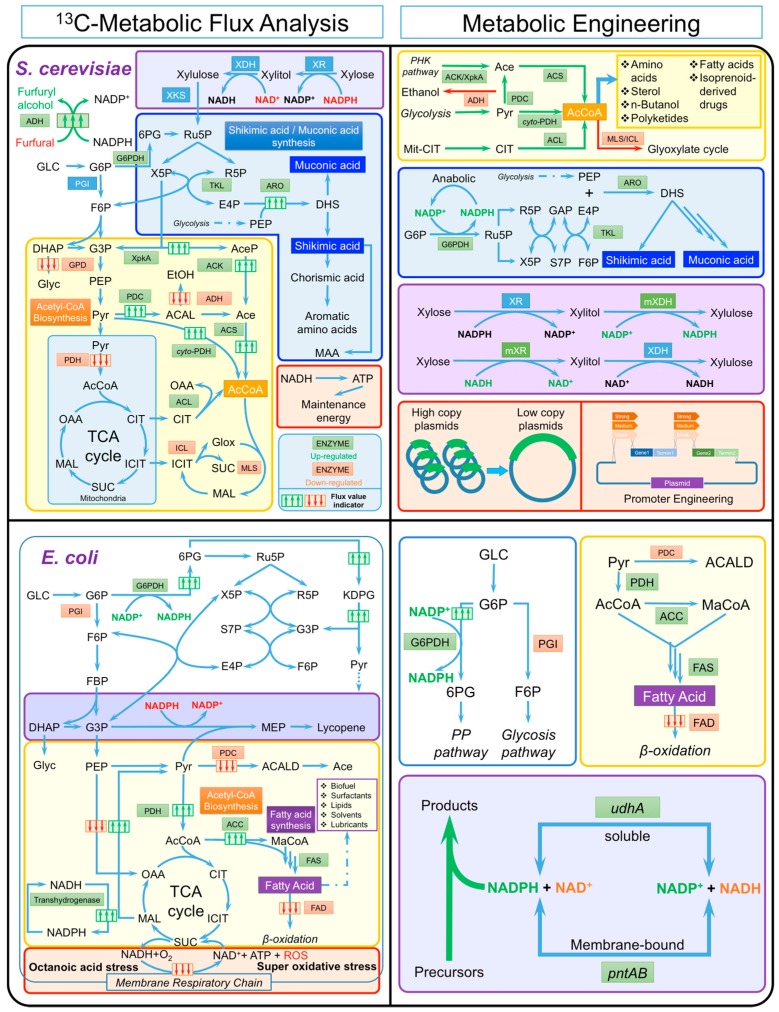
Integration of the major discoveries from ^13^C-MFA and corresponding metabolic engineering strategies for *S. cerevisiae* and *E. coli*. Abbreviations: G6P: Glucose 6-Phosphate, F6P: Fructose 6-phosphate, 6PG: 6-Phosphogluconate, Ru5P: Ribulose 5-Phosphate, X5P: Xylulose 5-phosphate, R5P: Ribose 5-Phosphate, E4P: Erythrose 4-Phosphate, PEP: Phosphoenolpyruvate, SA: Shikimic acid, MA: Muconic acid, MAA: Mycosporine-like Amino Acids, AA: Amino Acids. PGI: Phosphoglucose isomerase, G6PDH: G6P dehydrogenase, TKL: Transketolase, ARO: Pentafunctional protein ARO1p. DHAP: Dihydroxyacetone phosphate, G3P: Glyceraldehyde 3-phosphate, Glyc: Glycerol, AceP: Acetyl-P, EtOH: Ethanol, Pyr: Pyruvate, ACAL: Acetaldehyde, Ace: Acetate, AcCoA: Acetyl-CoA, OAA: Oxaloacetate, CIT: Citrate, ICIT: Isocitrate, SUC: Succinate, MAL: Malate, Glox: Glyoxylate, GPD: Glycerol-3-phosphate dehydrogenase. XpkA: Phosphoketolase, ACK: acetate kinase, PDC: Pyruvate Decarboxylase, ADH: Alcohol dehydrogenase, PDH: Pyruvate dehydrogenase, cyto-PDH: Cytosolic pyruvate dehydrogenase, ACS: Acetyl-CoA synthetase, ACL: ATP citrate lyase, ICL: Isocitrate lyase, MLS: Malate synthetase. MaCoA: Malonyl-CoA, ACC: Acetyl-CoA carboxylase, FAS: Fatty acid synthesis enzymes, FAD: Fatty acids degradation enzymes, MEP: 2-methylerythritol 4-phosphate, mXR: Mutated xylose reductase, mXDH: Mutated xylitol dehydrogenase.

The yeast *Saccharomyces cerevisiae*, an important microbial cell factory [73] and a model eukaryotic organism [24,74], has been widely used to produce various biofuels [8,75], bulk chemicals [50,76,77,78], pharmaceuticals [79,80], proteins, and other value-added products [81,82,83] by using various renewable feedstock. Although the knowledge on physiology of *S. cerevisiae* has been accumulated for over three decades, the complex metabolism in *S. cerevisiae* still impairs the improvement of chemical productivity in various bioprocesses. For example, the insufficient supply of several key precursors could be a bottleneck in biochemical production and the introduction of a heterologous sugar utilization pathway could lead to serious cofactor imbalance issues. In addition, high maintenance energy required by host cells will compete for the limited carbon source that keeps cells alive. Besides, various inhibitors in industrial fermentation process could dramatically inhibit cell growth, and even lead to cell death. To elucidate the complex yeast metabolism, ^13^C-MFA has been widely applied on *S. cerevisiae* to provide biological insights behind all above obstructions. Following these insights, metabolic engineers have developed versatile strategies to overcome rate-limiting steps for further improvement of biochemical production. In this section, as shown in Figure 3, we will summarize the findings from ^13^C-MFA and the corresponding metabolic engineering work for *S. cerevisiae*.

#### 3.1.1. Bottleneck Steps

Improving the supply of key precursors is one of the most effective strategies to boost the biochemical production in metabolic engineering. ^13^C-MFA analyzes the metabolic flux distribution in industrial microorganisms at steady state. By using ^13^C-MFA to elucidate the flux distributions of genetically perturbed cells, the bottleneck step in supplying key precursors for biochemical production can be identified. These elucidated major bottlenecks would provide direct instruction to design appropriate strategies for different biochemical production. For example, as a central metabolite, acetyl-CoA is a key precursor in the biosynthesis of sterols, amino acids, fatty acid-derived chemicals, polyketides, and isoprenoid-derived drugs [50]. To accommodate the cellular requirement, microorganisms use a variety of routes for acetyl-CoA synthesis (Figure 3). ^13^C-MFA has been applied to investigate the acetyl-CoA biosynthesis in *S. cerevisiae*. By comparing *S. cerevisiae* strains growing under purely oxidative, respiro-fermentative and predominantly fermentative conditions [84], the main pathway to supply cytosolic acetyl-CoA in *S. cerevisiae* was found to be the activated pyruvate bypass pathway. In this pyruvate bypass pathway, pyruvate is converted to acetaldehyde and then to acetate for synthesis of cytosolic acetyl-CoA. Although the pyruvate bypass pathway was activated, it was not strong enough to supply sufficient cytosolic acetyl-CoA when *S. cerevisiae* is engineered to produce acetyl-CoA-derived chemicals, such as n-butanol.

To enhance the cytosolic acetyl-CoA availability, various metabolic engineering strategies have been adopted in *S. cerevisiae* (Figure 3), including the disruption of competing pathways [50], such as malate synthetase (MLS1) and all alcohol dehydrogenases (ADH), as well as the introduction of heterologous biosynthetic pathways with higher catalytic efficiency and lower energy input requirement, such as cytosolic localized PDHs (cytoPDHs) [50] and ATP-citrate lyase (ACLs) [85]. A successful example of improving the biochemical production by enhancing acetyl-CoA supply is overexpressing the cytoPDHs, which led to 3-fold increase in n-butanol production in the engineered *S. cerevisiae* [50].

In addition to elucidating the bottleneck of cytosolic acetyl-CoA biosynthesis for wild-type yeast, ^13^C-MFA was also used to uncover the effect of a heterologous acetyl-CoA overproducing pathway, *i.e.*, phosphoketolase pathway (PHK), in a genetically engineered yeast strain with the expression of genes *xpkA* and *ack* from *Aspergillus nidulans* [51]. The PHK pathway was an alternative to the Embden-Meyerhof-Parnas (EMP) pathway for glucose dissimilation in several bacterial species [86] and filamentous fungi. For example, in *A. nidulans*, the utilization of this metabolic pathway increased carbon flow towards acetate and acetyl-CoA through the action of a phosphotransacetylase [87]. Flux distribution in the central metabolic pathways demonstrated the positive role of the PHK pathway on improving the supply of cytosolic acetyl-CoA in a *S. cerevisiae* strain, which was also accounted for the improved acetate production. Encouraged by this discovery, co-expression of the same PHK pathway and a wax ester synthase successfully improved the titer of fatty acid ethyl esters by 1.7 fold [88]. Such proof-of-concept study indicated that the PHK pathway could be established as a stand-alone route to divert flux from glycolysis to cytosolic acetyl-CoA supply, and holds great potential for improving the production of acetyl-CoA-derived chemicals in the future.

In addition to the central metabolite acetyl-CoA, several important precursors in the pentose phosphate pathway (PP pathway) also play a vital role in the synthesis of value-added compounds. For example, several chemicals, such as shikimic acid (a valuable drug precursor) and muconic acid (an important biopolymer precursor) could be produced from metabolites in the PP pathway [82]. To investigate the bottleneck in the PP pathway for the biosynthesis of shikimic acid, ^13^C-MFA was recently applied to four different engineered *S. cerevisiae* strains that were engineered to produce shikimic acids at different amounts (manuscript in preparation). By comparing flux distributions of the four strains, a higher flux through PP pathway was positively correlated with higher production of shikimic acid. This suggested that the low flux into the PP pathway, especially a limited supply of the precursor E4P from the PP pathway, could be the bottleneck for producing the shikimic acid. Indeed, it was found that when removing the original phenylalanine and tyrosine synthesis pathway, and overexpressing *aro1, aro4* and *tkl* genes to improve the metabolic fluxes in PP pathway (Figure 3), shikimic acid titer was increased by nearly 2-fold in *S. cerevisiae*. The similar strategy was applied to an engineered *S. cerevisiae* strain with a heterologous pathway for muconic acid synthesis. A 24-fold increase of muconic acid production has been achieved, compared to the initial strain containing only the muconic acid synthesis pathway [82].

#### 3.1.2. Cofactor Imbalance

Cofactors, e.g., NADH/NAD^+^ and NADPH/NADP^+^, play a key role as the redox carriers for catabolic and anabolic reactions, as well as important intermediates in cell energetics. However, the utilization of some cofactor-dependent production systems may lead to over-production or depletion of certain cofactors, and hence, break the original balances of cofactor usage in the cell metabolism. For example, one of the commonly used strategies to engineer *S. cerevisiae* for xylose utilization is introducing a fungal xylose pathway from native xylose-utilizing yeasts, such as *Pichia stipites.* Specifically, xylose could be converted to fermentable xylulose through the consecutive redox reactions catalyzed by NADPH-dependent xylose reductase (XR) and NAD^+^-dependent xylitol dehydrogenases (XDH), with xylitol produced as the intermediate (Figure 3). The usage of different cofactors for these two cofactor-dependent enzymes brings a notorious cofactor imbalance issue and severely restricts the xylose utilization in *S. cerevisiae*. More importantly, the cofactor imbalance issue is not standalone as shown by ^13^C-MFA, which is one of few analytical tools that can rigorously determine the cofactor usage in cell metabolism. It was found that the cofactor imbalance issue was intertwined with the central metabolism to induce network-level rewiring of carbon fluxes. Basically, ^13^C-MFA has been applied to systematically investigate xylose utilization of recombinant *S. cerevisiae* strains in an oxygen limited condition for ethanol production [35]. By implementing the ^13^C tracer experiments and running metabolic flux analysis for six recombinant strains with different origins of XR and XDH in the xylose pathway [35], the compensation of NADPH for the XR was supplied by the highly activated oxidative PP pathway. The strong activities in the TCA cycle were also observed and suggested that huge amounts of NADH needed to be consumed by oxidative phosphorylation. Concurrent with the global metabolic rewiring, only a small amount of the carbon fluxes was diverted to ethanol production.

Numerous efforts in metabolic engineering have been taken to solve the cofactor-imbalance issue in the xylose utilization of *S. cerevisiae*. One of the strategies is to engineer the cofactor dependent metabolic pathways that could decrease the xylitol production and enhance ethanol yield. For instance, lowering the flux through the NADPH-producing PP pathway could lead to increased ethanol yield and decreased xylitol production. This is attributed to an insufficient supplement of NADPH, which improves the NADH preference of XR, and thus, partially balance the cofactor usage of XR and XDH [89]. The other strategy used to tackle the cofactor imbalance issue is the partial alteration of the cofactor preference for these two enzymes, *i.e.*, altering the preference of XR to use NADH or altering XDH to use NADP^+^ as the cofactors, which would generate a cofactor-balance cycle for the initial two steps of xylose utilization to balance the cofactor usage (Figure 3).

The cofactor engineering strategy has proved to be functional in several studies. For example, by replacing the native *P. stipitis* XR with a mutated XR that has an increased preference of NADH, the ethanol yield was improved by ~40% with decreased xylitol production [90,91,92,93,94,95,96]. Similar successes have also been achieved in several other attempts to increase the NADP^+^ preference for the XDH, which successfully improved the ethanol production by 28%~41% [97,98,99,100]. In addition, by replacing the NADPH-producing PP pathway (glucose-6-phosphate dehydrogenase) with a fungal NADP^+^-dependent D-glyceraldehyde-3-phosphate dehydrogenase (NADP-GAPDH), no carbon would be lost when producing NADPH for the XR, which would provide more carbons for the ethanol production [101]. Also, improving the NAD^+^ regeneration directly by introducing the heterologous genes could increase the xylitol consumption by XDH and improve the ethanol production by obtaining more carbons [102]. Another efficient approach for the xylose fermentation is to use xylose isomerase, which could convert xylose to xylulose without the requirement of cofactors; and hence, using xylose isomerase completely bypasses the cofactor imbalance issue. However, as shown by a recent ^13^C-MFA study, the lower activity of glycolysis that led to inefficient re-oxidation of NADH could be a new bottleneck step when using xylose isomerase in *S. cerevisiae* [103].

#### 3.1.3. Metabolic Burden and Microbial Stress

Metabolic engineering is frequently equated with the heterologous production of a series of recombinant proteins. Nowadays, a large number of heterologous pathways have been introduced into a host cell, such as *S. cerevisiae*, in order to produce non-natural products with multiple genes inserted, deleted, replaced, or overexpressed. As a result, multiple kinds of chemicals can be produced with the implementation of complex genetic modification. However, the productivity achieved is often unsatisfactory due to the severe metabolic burden from the heterologous protein overexpression, a biological function that could be energetically expensive during transcription and translation. Such an issue, however, has not yet been well studied in the field of metabolic engineering.

The metabolic burden of industrial microorganisms is often reflected as elevated cell maintenance energy of industrial microorganisms shown as the increased TCA cycle fluxes by ^13^C-MFA studies (Figure 3). For engineered *S. cerevisiae* strains, it has been found that several genetic modifications could lead to the elevation of the cell maintenance energy. First, by using ^13^C-MFA for xylose-utilization *S. cerevisiae* strains, the elevated fluxes of TCA cycle were observed compared with wild-type strains in order to provide more ATP for cell maintenance [35]. The similar metabolic rewiring was observed for a S-Adenosyl-L-methionine (SAM) producing *S. cerevisiae* strain [34]. To avoid the introduction of extra metabolic burden or to compensate such a burden, metabolic engineers have explored three strategies: medium optimization, low-copy plasmids, and promoter engineering. Optimization of cultural medium and fermentation condition, such as the supplement of extra nutrients, could potentially remove stresses (e.g., nutrient limitation) and hence compensate the requirement for cell maintenance energy. In addition, utilization of a high-copy plasmid may increase risk of plasmid instability and metabolic burden [104], because the protein overexpression requires tremendous amounts of building blocks and energy, which could jeopardize the normal cell growth and increase the metabolic burden. The last method mentioned here to decrease metabolic burden is adjusting the promoter strengths of various genes, which could balance the pathways and avoid the accumulation of certain toxic intermediates.

In addition to the negative impact of the genetic interruption, environmental stresses, such as physical heat shock [105] and chemical acidity [106], could also lead to a seriously negative impact for biochemical production. For example, *S. cerevisiae* has been selected as a workhorse for lignocellulosic biofuel production, which holds promises for a sustainable fuel economy. However, the stress factors from the toxic compounds in the processed lignocellulosic hydrolysates, e.g., weak acids, furans, furfural, and phenolic compounds [107], have hampered the economic feasibility of biofuels by impacting the physiology and viability of microbial cells. Thus, the identification of the intracellular metabolic responses of *S. cerevisiae* to these stress factors is the key to rationally improve their resistance to inhibitors and productivity of biochemicals [39]. Compared to other commonly used approaches such as transcriptomics and proteomics analysis, ^13^C-MFA could provide more intuitive elucidation of the metabolic rewiring under stress conditions.

For example, furfural could be reduced to the less-toxic furfuryl alcohol with weak intrinsic capability of *S. cerevisiae* (Figure 3). However, the holistic view of metabolic responses to furfural is still missing. To further investigate the quantitative metabolic responses of *S. cerevisiae* under the increasing concentrations of furfural, ^13^C-MFA has been applied for wild-type and several evolved furfural-resistant strains in micro-aerobic and glucose-limited chemostats [38]. By comparing the different flux distributions under the increasing concentration of furfural stress, it is revealed that NADH-dependent oxireductases, which catalyzed the reduction of furfural, were the main defense mechanisms at a lower concentration of furfural (<15 mM), while NADPH-dependent oxireductases became the major resistance mechanism at the high concentration of furfural (>15 mM). Due to this shift of the major resistant mechanism, the carbon flux of pentose phosphate pathway increased as the main physiological response to high concentrations of furfural, which indicated that the increase of NADPH supply was the key to help *S. cerevisiae* better resist furfural stress. Inspired by this discovery, metabolic engineers overexpressed several NADPH-dependent oxireductases, particularly ADH7 and YKL071W, and successfully increased furfural resistance in the parent *S. cerevisiae* by 200% [38].

### 3.2. Escherichia coli

Due to the simplicity and fast growth, *E. coli* has been widely studied and applied for synthetic biology and metabolic engineering to produce various chemicals, such as advanced biofuels and value-added pharmaceuticals, with continuous accumulation of both knowledge and experiences in metabolic engineering. Nevertheless, many of the physiological responses in both wild type and engineered *E. coli* strains are not explicit due to its intricate regulation and metabolism. ^13^C-MFA, as a powerful approach to demystify the metabolic rewiring, has been applied to *E. coli* strains for two decades. Various valuable biological insights, such as key bottleneck steps in fatty acid-derived chemical production, cofactor imbalance issues, and metabolic responses to stress factor, have been elucidated, which helped metabolic engineers to design strategies for further improvement of the biochemical production. In this section, as shown in Figure 3, we summarized the recent outcomes of ^13^C-MFA studies on *E. coli* and corresponding metabolic engineering strategies.

#### 3.2.1. Bottleneck Steps

Fatty acids and fatty-acid-derived chemicals are the precursors to produce transportation fuels and industrial chemicals including surfactants, solvents, and lubricants [56]. Several successful studies using microorganisms to produce fatty acids and fatty acid-derived chemicals have been achieved with the highest titer of 8 g/L fatty alcohol produced by engineered *Rhodosporidium toruloides* using sucrose as substrate [108]. *E. coli* is also considered as an excellent host for fatty acid production because of its fast growth, simple nutrient requirements, well-understood metabolism, and well-established genetic tools. In the wild type *E. coli*, only a small amount of free fatty acids are detectable under normal conditions, which indicates that several genetic modifications are necessary for *E. coli* to improve the fatty acid production*.* The native synthesis pathways of saturated fatty acid include the conversion of acetyl-CoA into malonyl-CoA catalyzed by ATP-dependent acetyl-CoA carboxylase, the transesterification of malonyl-CoA into an acyl carrier protein (ACP) catalyzed by malonyl-CoA ACP transacylase (*fabD*), and the cyclic chain elongation process [109].

Recently, various fatty acid over-producing strains have been created to use different strategies to boost the fatty acid production. However, most studies focus on engineering terminal enzymes in fatty acid biosynthesis pathways and little is known about the central metabolism responses to fatty acid production. To uncover the key bottleneck steps in fatty acid production, ^13^C-MFA has been performed by using an engineered fatty acid over-producing *E. coli* DH1 strain with the overexpression of *tesA*, and *fadR* genes as well as the knock-out of *fadE* gene [56]. Compared to the wild-type *E. coli* strain, the flux in the engineered strain was significantly diverted from acetate synthesis to fatty acid synthesis, which suggested that the increased supply of key precursors in the fatty acid synthesis played a crucial role to increase subsequent fatty acid synthesis. The flux of the pentose phosphate pathway also dramatically increased to compensate large amounts of reduction powers, mostly NADPH, for the fatty acid production in the engineered strain. Finally, since more carbon fluxes were required to supply cytosolic acetyl-CoA (the starting point of fatty acid biosynthesis), the flux of the anaplerotic pathway into the TCA cycle decreased 1.7-fold in the engineered strain. Overall, as indicated by ^13^C-MFA, the supply of fatty acid precursor, cytosolic acetyl-CoA, and the reduction power, NADPH, were recognized as the key bottlenecks in microbial engineering for fatty acid production.

Various engineering strategies have been suggested and explored (Figure 3) in order to improve fatty acid production. For example, to overcome the challenge of limited supply of the key fatty acid precursor, malonyl-CoA, the acetyl-CoA carboxylase was over-expressed to provide more malonyl-CoA, which successfully improved the production of fatty acids [110,111]. Besides the enhancement of upstream synthesis pathways, the downstream degradation pathway, namely, the fatty acid degradation pathway was removed by knocking out *fadE* in *E. coli*. Combined with the over-expression of *tesA* and *fabF*, the yield of fatty acids was increased by nearly 3-fold [112]. Similarly, co-expression *fabZ* and a thioesterase from *Ricinus communis* in a *fadD* (a key gene in fatty acid degradation) deletion mutant could enhance the fatty acid titer by nearly 3-fold [113]*.* In summary, the bottleneck of precursor supply identified by ^13^C-MFA has now been well addressed in metabolic engineering for fatty acid production. The insufficient supply of NADPH, another bottleneck uncovered by ^13^C-MFA, could be the next direction to further improve the fatty acid production and requires the attention of metabolic engineers.

#### 3.2.2. Cofactor Imbalance

The imbalance issue of NADPH has been founded in several engineered *E. coli* strains via ^13^C-MFA. For example, ^13^C-MFA has been used to analyze the cell metabolism in wild type and fatty acid over-producing *E. coli* strains. As revealed by ^13^C-MFA, the engineered strain requires excessive NADPH compared to the wild-type strain, *i.e.*, 255 units compared to 179 units NADPH when the flux of glucose uptake was normalized to 100 units. However, the sum of NADPH supplied from central metabolism could only reach 100 units [56], which is deficient by more than 150 units of NADPH. This clearly indicated that more NADPH was required to improve fatty acid production in *E. coli*. In order to compensate for the NADPH deficiency, the transhydrogenase pathway was activated in the engineered *E. coli* with flux increasing by 70% compared to that in wild type strains (*i.e.*, from 90 units to 153 units) for fatty acid biosynthesis.

Realizing the significance of cofactor balance, particularly the NADPH supply, in engineered *E. coli* strains, metabolic engineers have adopted various strategies to overcome this challenge to enhance biochemical production. One strategy is switching the specificities of glycolytic enzymes, e.g., GAPDH, from NAD^+^-dependence to NADP^+^-dependence. Such a switch increased the availability of NADPH by building a NADPH-producing glycolysis pathway, and further improved the NADPH-dependent lycopene production by ~100% [52]. Also, several genetic modifications to redirect the metabolic flux from the glycolysis pathway into the PP pathway have been performed to enhance NADPH supply. These genetic modifications overexpressed *zwf* that encodes glucose-6-phosphate dehydrogenase (G6PDH) [114,115,116], deleted *pfkA* and *pfkB* that encode the phosphofructokinase (PFK) [117], or deleted *pgi* that encodes phosphoglucose isomerase [118,119] (Figure 3). In addition, transhydrogenase [120,121] or NAD kinase [52] was overexpressed to further boost the NADPH availability in *E. coli.* Overall, following the metabolic rewiring identified by ^13^C-MFA, the availability of NADPH has been increased for biochemical production in *E. coli*.

#### 3.2.3. Metabolic Burden and Microbial Stress

Several strategies to reduce the metabolic burden in *E. coli* (mentioned in 3.1.3) were achieved to increase the biochemical productivity (Figure 3). For example, using low-copy plasmid in the engineered lycopene-producing *E. coli* strain could increase the cell density by approximately 24% compared to the engineered strain with high-copy plasmid [122], although the cell density of low-copy plasmid strain is still inevitably lower than the control culture. Similarly, the titer of lycopene in the *E. coli* with low-copy plasmid was 20% higher than that with high-copy plasmid. Another example of engineered *E. coli* strain to decrease metabolic burden is adjusting the promoter strengths of various genes in taxadiene pathways to improve the efficiency of taxadiene production in *E. coli* [10]. In general, promoter engineering was implemented to tune the expression level of two modules in the taxadiene pathway: a native upstream methylerythritol-phosphate (MEP) pathway forming isopentenyl pyrophosphate and a heterologous downstream pathway forming terpenoid. As a result, the minimal accumulation of an inhibitory intermediate compound for cell growth, indole, was achieved by expressing the upstream pathway in a very low level, which led to ~15-fold increase of the taxadiene production.

In addition to elucidating the metabolic responses to the genetic modification of engineered *E. coli* strains, several ^13^C-MFA studies have recently been performed on the metabolic responses to different environmental stresses on *E. coli*. First, ^13^C-MFA was applied to elucidate the metabolic responses of *E.coli* to octanoic acid stress [37] (Figure 3). A decreased flux in the TCA cycle and an increased flux in the pyruvate oxidative pathway for producing acetate were observed by comparing the flux distributions of stressed and unstressed *E. coli* strains. It was hypothesized that octanoic acid triggered the membrane disruption and led to NAD^+^ deficiency due to the destabilization of membrane-bound proteins, such as NADH dehydrogenase. The interrupted regeneration of NAD^+^ would repress several key NAD^+^ dependent pathways, such as the malate dehydrogenase pathways in the TCA cycle, and the pyruvate dehydrogenase pathway. In addition, the pyruvate pool shrank under octanoic acid stress condition, which could be attributed to the repression of the PdhR regulator that is highly sensitive to pyruvate in controlling the expressions of the PDH complex, NADH dehydrogenase II, and cytochrome *bo*-type oxidase [123]. Based on the ^13^C-MFA results, several possible strategies to further enhance the octanoic acid resistance were proposed, including the supplement of pyruvate in the medium and the replacement of NADH/NAD^+^-sensitive enzymes.

In addition to the acidity stress, superoxide stress is another commonly encountered stress factor, which promotes the excessive production of reactive oxygen species (ROS) that have detrimental effects on cell metabolism and other physiological activities[124]. By performing ^13^C-MFA for *E. coli* strain under paraquat-induced superoxide stress, the global flux redistribution of the central carbon metabolism was observed to resist the superoxide stress. First, the flux of oxidative PP pathway has been increased by ~2-fold, which indicated the increased NADPH requirement under the superoxide stress. In addition, the NADH production has been dramatically repressed by diverting the fluxes from the pyruvate dehydrogenase pathway into the pyruvate bypass pathway for acetate synthesis as well as from the TCA cycle into the glyoxylate shunt to reduce the NADH generation and accumulate α-ketoglutarate for protein synthesis. As a result, the NADPH/NADH ratio has been dramatically increased as the cellular responses under oxidative stress for other strains. As revealed by ^13^C-MFA, the overall mechanism to resist the oxidative stress was increasing the NADPH production as the preferred reduction power and decreasing the NADH production as the key electron transporter in cell respiration. This mechanism is due to the pivotal role of NADH in the generation of most of the endogenous cellular ROS during the cell respiration and the effectiveness of NADPH to maintain the reductive environment for cellular activities. Based on the flux analysis, several potential strategies to further improve the tolerance of oxidative stress were indicated, including overexpressing the *zwf* gene encoding the G6P dehydrogenases and the genes encoding the transhydrogenase to improve the NADPH supply.

### 3.3. Bacillus Subtilis

**Figure 4:**
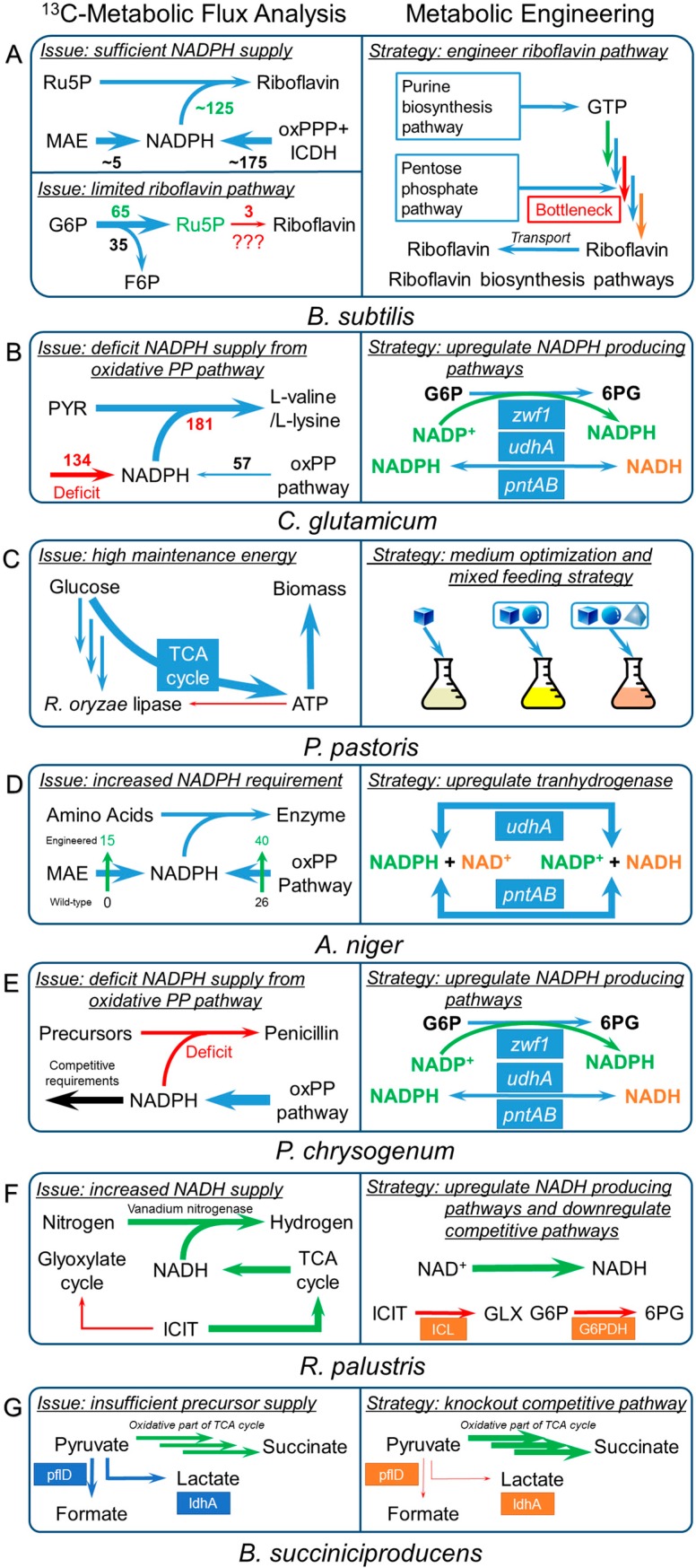
Integration of the key discoveries from ^13^C-MFA and the corresponding metabolic engineering strategies for (**A**) *B. subtilis*; (**B**) *C. glutamicum*; (**C**) *P. pastoris*; (**D**) *A. niger*; (**E**) *P. chrysogenum*; (**F**) *R. palustris*; and (**G**) *B. succiniciproducens*.

*Bacillus subtilis*, most well known as the microorganism engineered for commercial production of riboflavin [125], has been studied by ^13^C-MFA for both wild type and engineered strains in the past two decades [126,127]. Valuable insights of *B. subtilis* have been elucidated to better understand the cell metabolism and to further improve the biochemical production. The flux distributions of a riboflavin-producing *B. subtilis* strain has been rigorously investigated under three different dilution rates in chemostats [126]. As revealed by ^13^C-MFA, the activated PP pathway was observed, which not only supplied sufficient precursors but also produced sufficient NADPH (Figure 4A). More interestingly, the excessive NADPH production based on the flux analysis was observed under all the three dilution rates, especially in the low dilution rate without riboflavin production. In other words, the estimated amount of NADPH formation was found to be more than the NADPH requirements for both biomass and riboflavin production. The sufficient precursor supply from PP pathway could explain the high production of riboflavin and purine nucleotides. The transhydrogenase, which catalyzed the reversible conversion of NADPH to NADH, played an important role to re-oxidize the excessive NADPH that was generated due to the highly activated PP pathways.

A complex interplay between glycolysis and the TCA cycle through the pyruvate-OAA-PEP futile cycle was also observed, which provided the sufficient replenishment of metabolites in the TCA cycle [126,127]. As a result, the energy overproduction was found under different dilution rates, *i.e.*, the ATP formation estimated from the calculation was excessive compared to the ATP consumption. Considering that sufficient precursors, cofactors, and energy resources were supplied for the riboflavin synthesis in *B. subtilis*, the bottleneck for riboflavin production most likely resides in the biosynthesis pathways itself. In another large-scale ^13^C-MFA study on 137 null mutants of *B. subtilis* strains with different genetic backgrounds [128], a rigid flux distribution was observed, which is largely independent of the rate and yield of biomass. Thus, an unexpectedly stable metabolic state was maintained in *B. subtilis* under the given environment. This stable state was robust against the random genetic perturbations thanks to the sufficient flexibility of the acetyl-CoA branch point controlled by various transcriptional regulators. Therefore, *B. subtilis* could be considered as a robust host microorganism for biochemical production, with the proper extent of genetic perturbations.

Despite the advantages of using *B. subtilis* for biochemical production, ~50% increased requirement of maintenance energy caused by the genetic modification for riboflavin production was revealed by ^13^C-MFA, which could decrease the riboflavin productivity [129]. In addition, by implementing ^13^C-MFA for 8 strains of *Bacillus* species, including *B. subtilis* wild type, *spo0A*, *sigE* mutants and *natto*, *B. licheniformis* T218a, *B. licheniformis* T380B, *B. amyloliquefaciens* and *B. pumilus*, it was found that the maintenance energy required by *B. subtilis* was 35%~50% more than what was required by the other strains. However, consistent with the results of previous ^13^C-MFA studies [126,130], the excessive NADPH production was only observed in *B. subtilis* in contrast to other *Bacillus* strains with either balanced or even insufficient NADPH production (Figure 4A). This study emphasized another potential bottleneck in riboflavin production: the high maintenance energy. Since maintenance energy could be compensated by increasing ATP production from an elevated TCA cycle, the strategies mentioned in 3.1 and 3.2 to reduce or compensate the maintenance energy could also be applied to *B. subtilis* for improving biochemical production.

### 3.4. Corynebacterium Glutamicum

*Corynebacterium glutamicum,* the industrial workhorse for producing amino acids, such as L-lysine and L-valine, has been considered an important microorganism with extensive ^13^C-MFA studies [45,131,132,133,134]. Based on the insightful information provided by ^13^C-MFA, many metabolic engineering strategies have been developed to improve the amino acids production. For example, L-lysine is one of the major products of *C. glutamicum*, which is synthesized from the pyruvate and oxaloacetate requiring 4 moles NADPH to synthesize 1 mole L-lysine. By implementing ^13^C-MFA studies for L-lysine producing *C. glutamicum* strains, it was observed that the PP pathway was increased to supply NADPH, and the anaplerotic carboxylation pathway was enhanced to provide enough precursors for L-lysine synthesis [132,133,135] (Figure 4B). Following the discoveries from ^13^C-MFA studies, various metabolic strategies have been developed to improve the L-lysine production. First, the insufficient NADPH supply was overcome by overexpressing the enzymes in PP pathway, such as glucose-6-phosphate dehydrogenase [136], transketolase, and transaldolase [137], as well as 1, 6-bisphosphatase [138], to redirect fluxes towards PP pathway to improve lysine production. In addition to the overexpression of the native existed enzymes in PP pathway for the NADPH production, alteration of cofactor specificity of GAPDH from NAD^+^-dependence to NADP^+^-dependence successfully improved the lysine production by ~50% without decreasing cell growth rate [139]. To further investigate the responses of such alteration, ^13^C-MFA was performed to compare the flux distributions between wild type and mutated strains. It was found that the mutated GAPDH pathway was the major source of the NADPH in the mutated strain with the similar PP pathway flux, but with higher lysine production. Such discovery was consistent with the expectations, and demonstrated that ^13^C-MFA could indeed rationally guide the metabolic engineering and improve the microbial performance. In another ^13^C-MFA study, a strong increase in the PP pathway flux was found to be associated with L-valine production in a pyruvate decarboxylase deficient *C. glutamicum* strain, which again indicated that the NADPH supply was the key issue in L-valine production. By cloning a transhydrogenase from *E.coli* to enhance NADPH supply in *C. glutamicum*, L-valine yield was improved by ~200% [53].

### 3.5. Other Industrial Microorganisms

Although the above model microorganisms (e.g., *S. cerevisiae* and *E. coli*) have been well studied for a relatively long time with a better understanding of the cell metabolism, various non-model microorganisms have been recently highlighted for biochemical production due to their unique advantages. For example, *Pichia pastoris* has been used as an industrial microorganism to produce recombinant protein for more than three decades [140,141,142,143], due to the high cell density in growth, the strong and tightly regulated promoters, and partially functional post-translational modification [144,145]. Similarly, *Aspergillus niger* is another preferable industrial producer of various extracellular enzymes and antibiotics due to the advantages for protein secretion and post-translational modification [146]. *Penicillium chrysogenum* has been used to produce the penicillin G as a secondary metabolite of *P. chrysogenum*, which has been used as a narrow spectrum penicillin antibiotic against gram-positive organisms for more than 60 years [147,148]. In addition, *Rhodopseudomonas palustris*, a photosynthetic bacterium, could metabolize acetate and produce hydrogen at the non-growth stage when starved for nitrogen. Many studies also attempt to use some novel non-model microorganisms for producing versatile chemicals. For instance, *Basfia succiniciproducens* is found to be a novel producer with a high yield of succinate, a key platform chemical. By specifically revising the universally existing central metabolism model for various microorganisms, ^13^C-MFA has been performed for these non-model microorganisms. Here, as shown in Figure 4, we summarized multiple ^13^C-MFA studies on these non-model microorganisms that identified various issues related to biochemical production and listed the corresponding metabolic engineering strategies to solve these issues.

#### 3.5.1. *Pichia Pastoris*

^13^C-MFA has been used for study metabolism of *Pichia pastoris* engineered for the production of various heterologous proteins [64,65,66,67]. Two groups of *P. pastoris* strains were developed and grown under the mixed methanol and glucose culture: control group that expressed a mock plasmid, and mutant group that expressed either a low-copy or a high-copy plasmid to express *R. oryzae* lipase. By comparing the flux redistributions between control group and mutant group, it was found that the TCA cycle fluxes of both protein-expressing *P. pastoris* strains were much higher than the control strain in order to produce more ATP to sustain cell growth (Figure 4C). This elevated TCA cycle confirmed that the protein folding and conformational stress indeed imposed a metabolic burden on the microbial host. It was also found that using mixed methanol and glucose culture could reduce the fluxes in TCA cycle for the reference strain with the mock plasmid, compared to using glucose as a single carbon source. Such a decreased TCA cycle indicated that the mixed feeding strategy could compensate the high cell maintenance requirements and reduce part of the metabolic burden. Indeed, in another example, by using several novel feeding strategies to cultural SAM-producing *P. pastoris*, the production of SAM was found to be improved by ~35% [149,150]. Thus, reduction of the metabolic burden would be an efficient way to further improve the protein productivity in *P. pastoris.*

#### 3.5.2. *Aspergillus Niger*

*Aspergillus niger* is used for the recombinant production of the glycosylated enzyme fructofuranosidase, a biocatalyst of commercial interest for the synthesis of pre-biotic sugars that could induce the growth or activity of microorganisms to contribute to the well-being of humans. ^13^C-MFA has been applied to the recombinant strain *A. niger* SKAn1015, which expressed the fructofuranosidase encoding *suc1* gene and secreted 45 U/mL of the target enzyme, and its parental strain, SKANip8, which did not produce the target enzyme [151]. By comparing the flux distributions in the wild-type and recombinant strain, significant redirections of metabolic fluxes were observed in the recombinant strain as below: (1) more than a 50% increase of the oxidative PP pathway (oxPP pathway); (2) activated mitochondrial malic enzyme pathway (Figure 4D); and (3) more than a 60% decrease of TCA cycle*.* The increased oxidative PP pathway and activated malic enzyme pathways resulted in a relative increase of 43% for the NADPH supply as compared to the wild type. The similar increase of NADPH supply in recombinant *A. niger* strain was also observed in other ^13^C-MFA studies [152]. Based on ^13^C-MFA studies, the high flexibility of the PP pathway and the TCA cycle was found to cope with different environmental and intracellular perturbations [151]. Thus, to further improve the heterologous enzyme production, compensation of NADPH production and cell growth could be a new focus for metabolic engineering of *A. niger*.

#### 3.5.3. *Penicillium Chrysogenum*

Another example of ^13^C-MFA for non-model microorganisms is to elucidate the metabolic rewiring of *P. chrysogenum* in C-limited chemostats for penicillin-G production [153]. The significant differences in flux distribution through the central metabolic pathways were observed for each individual carbon source used, *i.e.*, glucose, ethanol, and acetate, but no significant changes were found for penicillin-G production. However, when using xylose and/or nitrate as carbon and/or nitrogen source, which required additional NADPH supply, the penicillin-G production decreased (Figure 4E). This interesting metabolic rewiring showed that the potential bottleneck of penicillin production probably resided on the primary metabolism, especially the NADPH production, but not so much on precursor supply due to the similar productivity under different substrates.

#### 3.5.4. *Rhodopseudomonas Palustris*

^13^C-MFA has also been applied to study *R. palustris,* a photosynthetic bacterium producing hydrogen at the non-growth condition starved for nitrogen, to elucidate the metabolic activities of the hydrogen producing and nonproducing conditions [154]. By tracking the ^13^C-labeled acetate through the central metabolism pathways, a shift of the acetate metabolism has been found, *i.e.*, under the hydrogen producing condition, the cell prefers to use the TCA cycle, instead of the glyoxylate cycle, to metabolize acetate. This metabolic rewiring provides more reduction power, *i.e.*, NADH, for hydrogen production by fully oxidizing the acetate. Therefore, improvement of NADH supply could contribute to enhancing the hydrogen production in *R. palustris* (Figure 4F)*.*

#### 3.5.5. *Basfia Succiniciproducens*

A ^13^C-MFA study was finished on *Basfia succiniciproducens* to improve the succinate production [155]. As elucidated by the ^13^C-MFA study, the metabolic flux distribution of the wild-type *B. succiniciproducens* strain with a high yield of succinate (0.75 mol/mol glucose) uncovered the parallel *in vivo* activity of the oxidative and reductive branch of the TCA cycle in *B. succiniciproducens*. In addition, it revealed two undesired pathways catalyzed by pyruvate-formate lyase (PflD) and lactate dehydrogenase (LdhA), which consumed the precursor pyruvate for succinate production (Figure 4G). Guided by the discovery of the ^13^C-MFA study, a doubly deletion Δ*pflD* Δ*ldhA B. succiniciproducens* strain was developed by genetic modification and achieved 45% increase of succinate yield.

## 4. Perspectives of Integrating ^13^C Metabolic Flux Analysis with Metabolic Engineering

**Figure 5:**
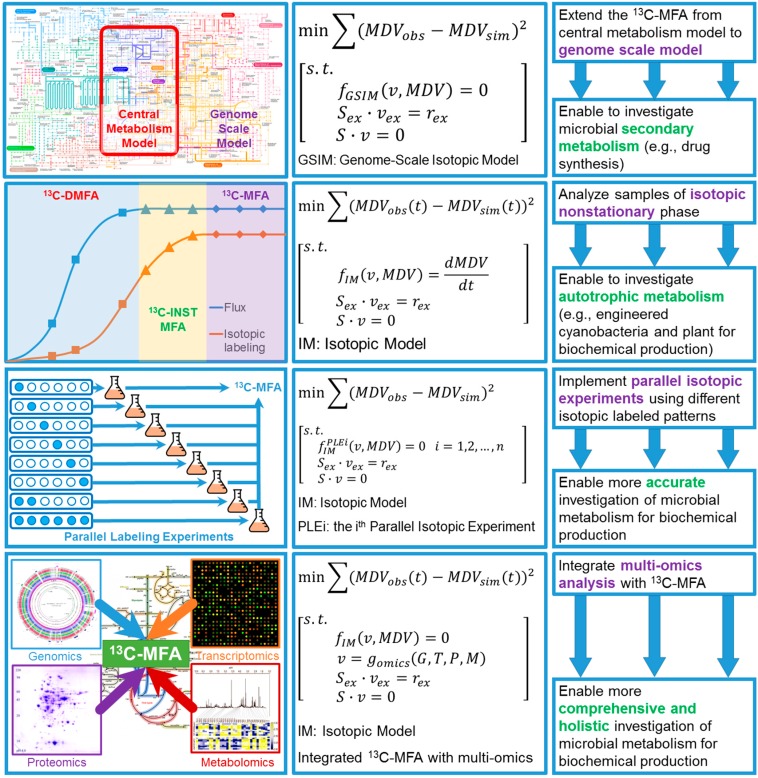
Recent advances and perspectives of ^13^C-MFA, including diagrams (left column), mathematical representation (middle column), and detailed descriptions of recent advances of ^13^C-MFA (right column).

In summary, ^13^C-MFA, as a flexible and powerful approach to elucidate the intracellular metabolic rewiring, has been widely performed for both model and non-model microorganisms, and unraveled numerous metabolic “mysteries” inside the wild type, evolved, and engineered strains. These biological insights uncovered by ^13^C-MFA have profound impact on the rational design of metabolic engineering strategies to further improve the biochemical production. However, several limitations are still restricting the accuracy and flexibility of ^13^C-MFA. For example, the traditional ^13^C-MFA can only be applied at metabolic and isotopic steady states [58], which could be difficult to use when the target chemicals are produced in non-steady states (e.g., drug synthesis in the stationary growth phase). In addition, most of the conventional ^13^C-MFA studies can only be applied in central metabolism [156], which has very limited use when analyzing the secondary metabolism of microorganisms. To overcome these challenges, novel experimental and computational methods have recently been developed to empower ^13^C-MFA studies. In this section, we will summarize recent breakthroughs in ^13^C-MFA and provide a perspective for novel routes to achieve the integration of ^13^C-MFA and metabolic engineering. The details of each novel approach for ^13^C-MFA, including graphical flowchart, mathematical representation, and brief descriptions of recent advances of ^13^C-MFA were shown in Figure 5.

### 4.1. Expand ^13^C-MFA into Genome Scale

The conventional ^13^C-MFA was only able to be used for the determination of flux distribution in the central metabolic network, mainly because of the difficulties in (1) measuring the isotopic labeling of the numerous low-abundant metabolites to provide more information needed for expanding the model; (2) providing a high quality genome-scale model along with the detailed atom maps of every reaction in the network; and (3) the huge computational burden of simulating isotopic labeling of all metabolites in genome-scale metabolic networks. However, with the continuous efforts committed to overcome these obstacles, several successful studies have been reported to extend the ^13^C-MFA to genome-scale. Thanks to the rapid development of high-resolution mass spectrometry, the accurate measurement of isotopic labeling of low-abundant metabolites became possible [157,158,159]. In addition, a novel algorithm known as Canonical Labeling for Clique Approximation (CLCA) [160] has been developed to provide the atom mapping information for the reactions of genome-scale metabolic model. By using this algorithm, a new bioinformatics database, MetRxn database [161], has been built and continues to be developed, which currently contains atom mapping information for over 27,000 reactions from 112 metabolic models [162]. To use this huge metabolic network with atom mapping information for the ^13^C labeling patterns, an efficient network decomposing method, the elementary metabolite units (EMU) method [68], was used to decrease the computational burden by 1~2 orders of magnitude for the genome-scale mapping model.

Recently, the first ^13^C-MFA study based on refined genome-scale mapping model (GSMM) of *E. coli* strain has been reported, which showed several advantages when using the genome-scale model instead of the central metabolic model [162]. In short, using the GSMM to elucidate the flux distribution via ^13^C labeling patterns obtained a better prediction of ^13^C-labeling patterns, resolved the alternative pathways in secondary metabolism for some key metabolites, and refined the cofactor and energetic metabolism. Therefore, genome-scale ^13^C-MFA could provide a more thorough investigation of microbial metabolism for biochemical production. This valuable information could guide the development of rational metabolic engineering strategies in a similar way that has been used for improving chemical production.

### 4.2. Isotopic Nonstationary ^13^C-MFA (^13^C-INST-MFA)

^13^C-INST-MFA was recently developed [163,164,165,166,167] to enable the application of ^13^C-MFA for various autotrophic systems including cyanobacteria and plants. In brief, other than collecting ^13^C-labeling patterns at the isotopic steady state, ^13^C-INST-MFA tracks the dynamics of ^13^C-labeling in intracellular metabolites and applies computational algorithms to calculate the steady-state metabolic fluxes that can best fit the ^13^C-labeling kinetics. ^13^C-INST-MFA has been performed to determine the autotrophic metabolism of *Synechocystis* sp. PCC6803 [168] and *Arabidopsis thaliana* [169]. Such autotrophic metabolism is unable to be elucidated via the conventional ^13^C-MFA because all of the metabolites will be universally labeled at the isotopic steady state when the autotrophic organisms are fed with ^13^CO_2_ and the information about pathway usage is completely lost. The merit of ^13^C-INST-MFA for metabolic engineering is the capability to rigorously determine the metabolisms of numerous autotrophic systems, which used to be mysterious. Since various autotrophic systems are promising cell factories [170,171,172] that convert inorganic carbon sources, e.g., CO_2_, into valuable chemicals, we can envision that ^13^C-INST-MFA could guide metabolic engineers to rationally modify such systems for improving the production of chemical products.

### 4.3. ^13^C-Based Dynamic Metabolic Flux Analysis (^13^C-DMFA)

^13^C-DMFA has recently been developed as an approach to investigate microbial metabolism at the metabolic non-steady state [29]. One of the proof-of-concept studies for ^13^C-DMFA investigated the *E. coli* metabolism in a fed-batch fermentation process for overproduction of 1,3-propanediol. By introducing several additional parameters to describe the fed-batch fermentation process, a time-resolved flux distribution map was obtained. Based on this time-resolved flux map, it is found that the intracellular flux associated with PDO pathway increased by 10% and the split ratio between glycolysis and pentose phosphate pathway decreased from 70/30 to 50/50. ^13^C-DMFA has provided a way for metabolic engineers to elucidate the dynamic metabolism during industrial fermentation, especially the fed-batch fermentation. The extension of ^13^C-DMFA to study microbial metabolism at the stationary growth phase is also expected since numerous high-value secondary metabolites are often produced during the stationary growth phase. With the insightful information about the metabolic rewiring at non-steady states, metabolic engineers could develop more appropriate strategies to improve the biochemical production, particularly microbe-based drug production.

### 4.4. Improve Accuracy of ^13^C-MFA via Parallel Labeling Experiments (PLE)

Another recent advance in ^13^C-MFA is the implementation of parallel labeling experiments (PLE) by using multiple isotopic tracers to track the cell metabolism, which has been proved to improve the flux estimation and observability of ^13^C-MFA [173,174,175]. Parallel labeling experiments have been combined with the rapid development of the high-throughput measurement and high-performance computational algorithms [173,174,175]. The unique advantages of PLE to the conventional ^13^C-MFA [176] are the improved accuracy of flux estimation [173,174] and reduced time for labeling experiments. With more accurate measurement of intracellular carbon fluxes provided by PLEs, the higher resolution of microbial metabolism will be offered to metabolic engineers in the near future to develop fine-tuned engineering strategies in improving biochemical production.

## 5. Conclusions

Metabolic engineering has been rapidly developed for various industrial microorganisms to produce bulk chemicals, fuels, and drugs from renewable feedstock, in order to free the modern society from the depleting fossil fuel feedstock. However, the complex microbial metabolism is one of the most challenging obstacles for metabolically engineered microorganisms to reach an industrially satisfactory yield, titer or productivity. In order to overcome this challenge, ^13^C-MFA has been continuously developed and successfully applied to assist the rational design of metabolic strategies for both model and non-model microorganisms by rigorously quantifying the carbon flux distribution in central metabolism. As shown in this review, multiple issues in biochemical production, such as bottleneck pathways in biochemical synthesis, cofactor imbalance issue in host cells, and energetic requirements in cell maintenance, have been revealed by ^13^C-MFA, which guides the development of the appropriate metabolic engineering strategies for successful improvements of the target chemicals to different extents. To further advance ^13^C-MFA, several techniques have been recently developed to demystify the secondary metabolism, improve the accuracy and resolution of the flux distribution, and investigate the metabolism for autotrophic organisms as well as the microbial metabolism at the metabolic non-steady state. We believe that, by using ^13^C-MFA for various cell factories, metabolic strategies will be more rationally designed and successfully applied to improve the biochemical production.
